# Linking minerals to bacterial genes

**DOI:** 10.1093/nsr/nwac265

**Published:** 2022-11-23

**Authors:** Shucheng Xie

**Affiliations:** State Key Laboratory of Biogeology and Environmental Geology, China University of Geosciences (Wuhan), China

The biogeochemical roles of microbes in the Earth system are relatively well known [1], but their biogeophysical roles in Earth history are yet to be investigated. Magnetotactic bacteria (MTB) may have been the earliest organisms to sense Earth's magnetic field and to form intracellular nanocrystals of magnetic minerals, so-called magnetotaxis and magnetosomes, respectively [2]. These magnetosome minerals are highly resistant to diagenesis and generally well-preserved in sediments or sedimentary rocks as magnetofossils [3], making excellent archives of the biogeophysical activity of microbes. Investigations of magnetosome biogenesis and assembly in MTB are critical not only for understanding the mechanism of biomineralization and the evolution of magnetoreception in organisms, but also for probing magnetofossil records as reliable proxies for paleomagnetic and paleoenvironmental analyses. However, such research remains limited to very few cultured bacterial strains, even though MTB were discovered over half a century ago [4,5].

MTB are a group of morphologically, phylogenetically and metabolically diverse prokaryotes [6], affiliated with at least four different bacterial phyla, each of which may have group- or even species/strain-specific magnetosome crystal morphologies [7]. However, most progress in understanding magnetosome formation relies on only two genetically tractable strains affiliated with *Magnetospirillum* of Alphaproteobacteria: *M. magneticum* AMB-1 and *M. gryphiswaldense* MSR-1 [5,8]. Considering the phylogenetic diversity of uncultured MTB and their diverse magnetic crystal morphologies and chain assemblies, a general model for gene networks that control or regulate magnetic particle biogenesis and chain assembly needs to be established on the basis of all available cultured and uncultured data.

To solve this problem, Jinhua Li and co-workers developed a general strategy for identifying and characterizing uncultured MTB from natural environments at the single-cell level [9]. In recent work by Liu *et al*. [10], they further integrated metagenomics to create a more robust methodology for the genomic and phenomic study of uncultured and cultured MTB. They generated new data sets of genomic and magnetosome morphological information for 15 uncultured MTB strains. Combined with 32 data sets from previously well-characterized cultured or uncultured MTB strains, they have assembled the largest genomics-magnetosome association database available to date.

This study links, apparently for the first time, the production of magnetic minerals to specific genes in MTB at the species/strain level, building on earlier observations that the genes associated with magnetosome formation (i.e. magnetosome gene clusters, MGCs) vary among different MTB clades [6]. This innovative study therefore provides critical evidence for construction of a general model for gene networks that control/regulate magnetosome biomineralization and assembly in MTB. Its key findings include:

First, confirmation of the core genes (i.e. *mamABEIKMPQ*) and the phylum-specific genes (e.g. MACGPs, *mad* and *man*) related to magnetosome formation. Interestingly, the genes *mamL* and *mamO*, which were previously thought to be core genes [6], are absent in the phyla Nitrospirota and Desulfobacterota, respectively. In some MTB, *mad* and *man* genes may be involved in the growth of bullet-shaped magnetosome magnetite. These findings provide genetic evidence for the phylum-specific morphology of magnetosomes, which indicates that magnetofossil crystal morphology from the ancient geological record has the potential to be a reliable proxy for the taxonomic lineage of ancient MTB and their paleoecology.

Second, demonstration of the key role of *mamK* in magnetosome chain assembly [6,9], suggesting that some group-specific magnetosome genes (e.g. *mamJ, mamY, mcaAB* and *mad28*) are responsible for diverse chain configurations among MTB clades. For instance, the copy number, arrangement and homology of the *mamK* gene may influence the magnetosome chain structures in magnetotactic Pseudomonadota, and the Mad24 and Man5 genes may be involved in the assembly of multiple magnetosome chains in MTB of the Desulfobacterota and Nitrospirota phyla, respectively. This study further proposes a general model for the gene networks that control/regulate magnetosome biomineralization, including magnetosome membrane formation, protein sorting, iron transportation and magnetite nucleation, crystal mineralization, and chain assembly, in diverse MTB clades. This finding will further our understanding of magnetosome biomineralization in multiple bacterial clades and could pave the way for use of magnetofossils in paleoecology, paleoenvironment and evolution research.

Yet, the general model proposed here remains incomplete and will require refinement. Further investigation of more phylogenetically diverse MTB is needed in order to achieve a deep understanding of magnetosome biomineralization at the molecular level. Nonetheless, the new results of Jinhua Li and co-workers significantly expand our knowledge of the genetic basis for magnetosome biomineralization and will likely serve as a guiding framework for future molecular-scale studies of biogenesis and chain assembly of magnetosomes. The linkage of minerals to genes in MTB systems achieved in this study offers an excellent example for other fields of geomicrobial research.
